# Awareness and acceptability of human papillomavirus vaccine: an application of the instrumental variables bivariate probit model

**DOI:** 10.1186/1471-2458-12-31

**Published:** 2012-01-13

**Authors:** Young Kyung Do, Ker Yi Wong

**Affiliations:** 1 Program in Health Services and Systems Research, Duke-NUS Graduate Medical School Singapore, Singapore

**Keywords:** Preventive medicine, Sexually transmitted infections, Gynecology

## Abstract

**Background:**

Although lower uptake rates of the human papillomavirus (HPV) vaccine among socioeconomically disadvantaged populations have been documented, less is known about the relationships between awareness and acceptability, and other factors affecting HPV vaccine uptake.

The current study aimed to estimate the potential effectiveness of increased HPV vaccine awareness on the acceptability of HPV vaccination in a nationally representative sample of women, using a methodology that controlled for potential non-random selection.

**Methods:**

This study used a population-based sample from the 2007 Health Information National Trends Survey, a cross-sectional study of the US population aged 18 years or older, and focused on the subsample of 742 women who have any female children under the age of 18 years in the household. An instrumental variables bivariate probit model was used to jointly estimate HPV vaccine awareness and acceptability.

**Results:**

The proportion of HPV vaccine acceptability among the previously aware and non-aware groups was 58% and 47%, respectively. Results from the instrumental variables bivariate probit model showed that the estimated marginal effect of awareness on acceptability was 46 percentage points, an effect that was even greater than observed.

**Conclusions:**

Among populations who are not currently aware of the HPV vaccine, the potential impact of raising awareness on acceptability of HPV vaccination is substantial. This finding provides additional support to strengthening public health programs that increase awareness and policy efforts that address barriers to HPV vaccination.

## Background

Human papillomavirus (HPV) and related diseases are major public health problems in the United States. Approximately 20 million Americans are currently infected with HPV while another 6 million are newly infected each year [1]. In 2010, an estimated 12,200 new cases of invasive cervical cancer - the most severe consequence of HPV infection - were diagnosed, and 4,210 women died from cervical cancer [2]. Over the past decades, a steady decrease in the cervical cancer incidence and mortality has been reported [2]. Still, cervical cancer disproportionately affects poor minority women [3]; along with increased incidence of cervical cancer among female population groups with lower education and income, and higher poverty [4,5], substantial disparities in stage at diagnosis and mortality also remain by race/ethnicity [3-8].

The persistent, albeit, diminishing socioeconomic disparities in overall cervical cancer incidence and mortality, can be partly explained by the constantly lower screening rates among disadvantaged populations, despite the widespread acceptance of the Papanicolau (Pap) test [9-12]. The recent Food and Drug Authority (FDA) approval of HPV vaccines which prevent the most common HPV infections (Gardasil^® ^in 2006 and Cervarix^® ^in 2009) [13] has provided an additional opportunity to reduce the burden of cervical cancer. This medical breakthrough can also potentially reduce cervical cancer disparities since high-risk groups [3-5,14] are the ones expected to benefit the most from HPV vaccines. Yet, these high-risk groups are less likely to receive the Pap test [11]. Although HPV vaccines are widely regarded as a key preventive measure for the universal benefit of all women [15], early evidence of lower HPV vaccination rates among poor minority women [16-18] indicates that disparities persist even in HPV vaccination. It is therefore critical to better understand the underlying factors of the disparities in HPV vaccination to maximize the HPV vaccines' potential for reducing the racial/ethnic and socioeconomic disparities in cervical cancer.

Major barriers to HPV vaccine implementation among at-risk populations include high vaccination costs and lack of a usual source of care. Limited community involvement in stimulating awareness of cervical cancer and available screening methods is another important obstacle that needs to be overcome [15]. Awareness is usually the first stage in the process of adopting a particular preventive health behavior [19]. Previous studies from the US and the UK have found that racial/ethnic minorities and socioeconomic disadvantages were associated with lower levels of awareness of either HPV vaccination or HPV [16,20-24]. Acceptability of the HPV vaccine, however, was not necessarily lower among racial/ethnic minorities and socioeconomically disadvantaged groups in the US [22,25]. Among a multi-ethnic sample of 18-to-55-year-old women in Los Angeles County, California, Latinas and Asian/Pacific Islander women were more willing to be vaccinated than White or Black women, whereas education was inversely associated with the intention to become vaccinated [22]. Interestingly, the highest levels of HPV vaccine acceptability were reported among the least educated and poorest individuals, although most of the estimated differences by racial/ethnic and socioeconomic characteristics were statistically insignificant at the 5% significance level [26]. A systematic review on HPV vaccine acceptability also found that lower education levels were associated with higher acceptability and that racial/ethnic minority groups showed similar levels of acceptability [25]. These findings suggest that racial/ethnic minorities and socioeconomically disadvantaged population groups who are less likely to be aware of the HPV vaccine, can potentially show even higher levels of HPV vaccine acceptability if they were given adequate information. If true, this hypothesis has important implications for public health practice and prevention policy. It can offer another rationale for public health programs to further raise HPV vaccine awareness among racial/ethnic minorities and socioeconomically disadvantaged groups, and strengthen policy efforts to address other barriers to HPV vaccination.

Little attention, however, has been paid to the potential impact of improving HPV vaccine awareness on increasing HPV vaccine acceptability at a population level. Observed differences in HPV vaccine acceptability levels between the previously "aware" and "non-aware" groups, including results from standard regression models, can be misleading because the two groups may differ in many unobserved ways. If the aware group consists of more advantaged individuals who may be more (less) acceptable of vaccinations, a fair comparison in HPV vaccine acceptability between the aware and non-aware groups cannot be achieved because the difference in acceptability may overestimate (underestimate) the marginal effect of improved HPV vaccine awareness.

This study aimed to quantify the marginal effectiveness of increasing HPV vaccine awareness on acceptability, using a nationally representative sample of women who reported having any female children under the age of 18 in the household. The hypothesis to be tested is that HPV vaccine awareness increases vaccine acceptability and that this population-level estimate is greater when unobserved heterogeneity between the aware and non-aware groups is taken into account than otherwise. To this end, the current study exploited a unique survey question that captured hypothetical HPV vaccine acceptability, and employed a statistical method that controlled for unobserved differences between the two comparison groups.

## Methods

### Data

The present study used data from the Health Information National Trends Survey 2007 (HINTS 2007) (Health Communication and Informatics Research Branch, Division of Cancer Control and Population Sciences, National Cancer Institute) [27], a study of a civilian, non-institutionalized population in the US aged 18 years and older. The HINTS 2007 was conducted between December 2007 and April 2008. The 2007 survey started just over a year after the quadrivalent HPV-6/11/16/18 vaccine (Gardasil/Silgard^®^) was licensed in the US (June 2006); the bivalent HPV-16/18 vaccine (Cervarix^®^) was not FDA approved until October 2009 [13]. The sample population was surveyed using either of these two instruments: a computer-assisted telephone interview or a mailed questionnaire, with response rates of 24.23% and 30.99%, respectively. The HINTS focused on the public's access to and use of cancer-related information, including a set of questions that was specifically related to cervical cancer and the HPV. In the present study, the sample was restricted to women who reported having any female children under the age of 18 in the household (N = 880). This particular group was chosen because the Advisory Committee on Immunization Practices (ACIP) recommends routine HPV vaccination for 11- and 12-year-olds with catch-up vaccination up to 26 years old [1]. For females below 18 years old, parental consent was needed to authorize the HPV vaccination. Thus, parental awareness and acceptability of the HPV vaccine is a crucial factor in the utilization of HPV vaccination among these female adolescents. After excluding observations with missing values for the study variables, the final sample consisted of 742 women.

### Variables

Acceptability of HPV vaccination was defined using the survey question, "A vaccine to prevent the human papillomavirus or HPV infection is recommended for girls aged 11-12 and is called the cervical cancer vaccine, HPV shot, or GARDASIL^®^. If you had a daughter that age, would you have her get it?" Respondents were given the following options to choose from: "Yes", "No", and "Not sure/It depends". For the statistical analysis, a binary indicator variable was created using "Yes" (= 1) versus "No" and "Not sure/It depends" (= 0), as defined by Fang and colleagues [26]. A dichotomous variable for HPV vaccine awareness was defined using the survey question, "A vaccine to prevent HPV infection is available and is called the cervical cancer vaccine or HPV shot. Before today, have you ever heard of the cervical cancer vaccine or HPV shot?" In the HINTS survey, this question on HPV vaccine awareness (No. 61) preceded the question on acceptability (No. 74).

The regression analysis included the following covariates: age, race, household income, and education level; having any insurance, any regular health care provider, past medical history of cancer, and family history of cancer; and self-rated health status, and census division. The specific categories used are shown in Table 1 and four-category dummy variables were created for combined annual household income. Considering the observed frequencies and the categories used by Fang et al. (2010) [26], income categories from the original questionnaire were combined as follows: "$20,000 - < $35,000" and "$35,000 - < $50,000" in one category, and "$75,000 - < $100,000" and "$100,000 or More" in another. A respondent was regarded as *having a regular health care provider *if she responded positively to the question, "Not including psychiatrists and other mental health professionals, is there a particular doctor, nurse, or other health professional that you see most often?" Similarly, *having any insurance *referred to an affirmative response to the question, "Do you have any kind of health care coverage, including health insurance, prepaid plans such as HMOs, or government plans such as Medicare?" Health status of respondents were based on their responses to the question, "In general, would you say your health is excellent/very good/good/fair/poor." There were nine census divisions, covering the New England Census Division throughout the Pacific Census Division.

**Table 1:** Summary statistics of study variables

	All		Aware		Non-aware	
	**(N = 742)**		**(N = 629)**		**(N = 113)**	

	**Freq.**	**%**	**Freq.**	**%**	**Freq.**	**%**

Would have daughter get HPV vaccine	415	55.8	362	57.7	53	46.9

Aware of HPV vaccine	629	80.1	-	-	-	-

Age group						

18-34	262	44.8	221	44.1	41	47.9

35-39	142	20.2	121	19.2	21	24.1

40-44	137	16.6	118	18.3	19	10.2

45+	201	18.3	169	18.5	32	17.8

Race/ethnicity						

Non-Hispanic White	461	57.9	421	64.4	40	31.7

Non-Hispanic Black	115	17.3	91	16.5	24	20.8

Hispanic	103	16.9	69	12.1	34	36.2

Other non-Hispanic	63	7.9	48	7.2	15	11.4

Household income						

$75,000 or more	280	32.1	255	34.6	25	22.0

$50,000 - < $75,000	130	17.1	120	19.6	10	7.0

$20,000 - < $50,000	200	32.7	156	30.5	44	41.9

Less than $20,000	132	18.1	98	15.4	34	29.2

Education						

College graduate	288	24.2	262	27.1	26	12.8

Some college	250	40.2	227	44.4	23	23.6

High school graduate	139	22.8	105	21.1	34	29.8

Less than high school	65	12.7	35	7.5	30	33.8

Having any health insurance	621	82.0	542	86.7	79	62.8

Having a regular health care provider	539	70.1	473	74.6	66	54.7

Past medical history of cancer	57	4.2	54	5.0	3	1.2

Family history of cancer	562	73.8	490	77.1	72	60.2

Self-rated health status						

Excellent	97	13.7	87	13.9	10	12.6

Very good	267	33.9	241	36.1	26	25.6

Good	276	38.7	227	37.9	49	42.1

Fair	83	11.6	58	9.9	25	18.3

Poor	19	2.1	16	2.3	3	1.6

Heard about test for lung cancer	175	22.1	163	25.4	12	8.4

Heard about clinical trials	547	68.7	499	74.8	48	44.5

*Notes: *Frequency is unadjusted frequency and % is adjusted with sampling weights

### Statistical analysis

To address the issue of unmeasured differences between the aware and non-aware groups which may influence acceptability of HPV vaccination, the current study employed an instrumental variable (IV) bivariate probit model. This method has been applied in various public health and health economics research to address similar statistical issues [28-31]. In this study, the IV bivariate probit model estimated the following joint model of acceptability of HPV vaccination (Eq. 1) and awareness of the HPV vaccine (Eq. 2).

(1) $$Accept^{*}=\beta Aware+\gamma_{1}X+\varepsilon_{1}$$

(2) $$Aware^{*}=\gamma_{2}X+\delta Z+\varepsilon_{2}$$

where Accept* is a continuous latent variable for the indicator variable of HPV vaccine acceptability (*Accept *= 1 if Accept* > 0, otherwise 0) and Aware* is a continuous latent variable for the indicator variable of whether the respondent has heard of HPV vaccine (*Aware *= 1 if Aware* > 0, otherwise 0). *X *denotes a set of covariates common to Eq. 1 and Eq. 2, and *Z *denotes a set of IVs included only in Eq. 2. *β*, *γ_1_*, *γ_2_*, and δ are coefficients for corresponding variable(s) in the models, whereas *ε_1 _*and *ε_2 _*are error terms of Eq. 1 and Eq. 2, respectively.

It is possible that unexplained differences in HPV vaccine awareness in Eq. 2 (*ε_2_*) are not correlated with unmeasured factors in Eq. 1 (*ε_1_*) that influence HPV vaccine acceptability. In this case, estimating Eq. 1 alone would already provide a valid estimate of *β*, which will capture the extent to which being aware of the HPV vaccine leads to HPV vaccine acceptability. If *ε_1 _*and *ε_2 _*are correlated, however, consistent estimates can be obtained by jointly estimating the two equations in the bivariate probit model. A formal test can be conducted to examine the correlation coefficient (*ρ*) between *ε_1_*, and *ε_2_*. Estimating the IV bivariate probit model efficiently requires good IVs (*Z *in Eq. 2) that must satisfy two basic conditions, these IVs: (1) should predict the outcome of *Aware *and (2) should not directly affect the outcome of *Accept*. The HINTS 2007 contains such potentially good IVs because access to information related to the various types of cancers is available. For example, a respondent who gave a positive answer to the question, "Have you heard of any tests to find lung cancer before the cancer creates noticeable problems?", would be more likely to have also heard about the HPV vaccine. However, responses to this question would be unlikely to directly influence the extent to which the respondent has greater acceptability of HPV vaccination. In the same vein, a positive response to the question "Have you ever heard of a clinical trial?" would strongly predict a greater probability of HPV vaccine awareness but unlikely affect the level of HPV vaccine acceptability. The current study used these two IVs, after exploring other variables related to the access to different types of information on cancer. Tests for these underlying assumptions suggested that these IVs predict *Aware *statistically significantly but are not directly associated with *Accept *other than through *Aware*. Stata 11.0 (StataCorp LP, College Station, TX) was used in all statistical analyses, taking into account a complex survey design.

## Results

### Descriptive statistics

Acceptability of the HPV vaccine was 57.7% among women who were aware of the vaccine and 46.9% among those who were not aware (Table 1). The majority of women who were aware of the vaccine were Whites (64.4%), whereas the majority of women who were not aware belonged to the ethnic minority (68.4%): specifically 20.8% non-Hispanic Blacks, 36.2% Hispanics and 11.4% other non-Hispanics. Among women who were aware, 54.2% reported a household income of at least $50,000, and 71.5% had at least a college level education - these percentages were nearly twice the values reported among those who were not aware (29% and 36.4%, respectively). More women in the aware group reported having health insurance (86.7% vs. 62.8%), a regular health care provider (74.6% vs. 54.7%), a past medical history of cancer (5.0% vs. 1.2%) and a family history of cancer (77.1% vs. 60.2%) than women in the non-aware group.

### Impact of HPV vaccine awareness on the acceptability of HPV vaccination

In the probit model of acceptability in which the awareness variable was assumed to be exogenous (Table 2), awareness of the HPV vaccine was not found to be a statistically significant influence on the acceptability of the HPV vaccine (coefficient = 0.275, 95% C.I.: –0.135, 0.686) with the estimated marginal effect of a 10-percentage-point increase. On the other hand, results from the IV bivariate probit model in Table 3 showed that awareness of the HPV vaccine had a statistically significant and positive association with acceptability of the vaccine (coefficient = 1.384, 95% C.I.: 0.725, 2.044). Based on the estimated coefficients of the bivariate probit model, HPV vaccine awareness improved the acceptability of the vaccine by 46 percentage points. In both the probit and bivariate probit models, socioeconomic characteristics such as household income and having any health insurance, a regular health care provider, a past history and a family history of cancer were not found to be significantly associated with acceptability.

**Table 2:** Probit model of acceptability of HPV vaccine (N = 742)

	Coefficient	(95% C.I.)
Aware of HPV vaccine	0.275	(–0.135, 0.686)

Age group		

18-34	Reference	

35-39	–0.185	(–0.565, 0.194)

40-44	–0.187	(–0.558, 0.183)

45+	–0.041	(–0.378, 0.296)

Race/ethnicity		

Non-Hispanic White	Reference	

Non-Hispanic Black	–0.396†	(–0.828, 0.037)

Hispanic	–0.008	(–0.512, 0.497)

Other non-Hispanic	–0.476	(–1.212, 0.259)

Household income		

$75,000 or more	Reference	

$50,000 - < $75,000	–0.253	(–0.673, 0.167)

$20,000 - < $50,000	0.130	(–0.329, 0.590)

Less than $20,000	0.206	(–0.396, 0.808)

Education		

College graduate	Reference	

Some college	0.066	(–0.297, 0.429)

High school graduate	0.045	(–0.386, 0.476)

Less than high school	0.273	(–0.284, 0.830)

Having any health insurance	–0.137	(–0.627, 0.353)

Having a regular health care provider	0.220	(–0.156, 0.596)

Past medical history of cancer	0.215	(–0.249, 0.679)

Family history of cancer	0.146	(–0.122, 0.414)

Self-rated health status		

Excellent	Reference	

Very good	0.233	(–0.188, 0.655)

Good	0.474*	(0.005, 0.942)

Fair	0.246	(–0.265, 0.757)

Poor	0.006	(–1.038, 1.050)

Constant	–0.119	(–0.938, 0.699)

Notes: †p < 0.10, *p < 0.05, **p < 0.01. A set of dummy variables for survey regional division in the 2007 HINTS was included in the estimation but their coefficients are not presented here

**Table 3:** Bivariate probit model of awareness and acceptability of HPV vaccine (N = 742)

	Equation: Aware of HPV vaccine		Equation: Would have daughter get HPV vaccine	
	**Coefficient**	**(95% C.I.)**	**Coefficient**	**(95% C.I.)**

Aware of HPV vaccine	-		1.384**	(0.725, 2.044)

Age group				

18-34	Reference		Reference	

35-39	0.056	(–0.468, 0.579)	–0.164	(–0.568, 0.239)

40-44	0.123	(–0.455, 0.701)	–0.211	(–0.582, 0.161)

45+	–0.255	(–0.778, 0.267)	0.011	(–0.316, 0.337)

Race/ethnicity				

Non-Hispanic White	Reference		Reference	

Non-Hispanic Black	–0.410	(–0.935, 0.114)	–0.258	(–0.738, 0.222)

Hispanic	–0.401	(–1.097, 0.295)	0.177	(–0.391, 0.746)

Other non-Hispanic	–0.680†	(–1.416, 0.057)	–0.224	(–0.894, 0.445)

Household income				

$75,000 or more	Reference		Reference	

$50,000 - < $75,000	0.424	(–0.190, 1.037)	–0.300	(–0.718, 0.118)

$20,000 - < $50,000	–0.074	(–0.680, 0.533)	0.130	(–0.307, 0.567)

Less than $20,000	0.142	(–0.521, 0.806)	0.177	(–0.438, 0.792)

Education				

College graduate	Reference		Reference	

Some college	0.202	(–0.284, 0.688)	0.025	(–0.338, 0.388)

High school graduate	–0.306	(–0.881, 0.269)	0.147	(–0.249, 0.543)

Less than high school	–0.801*	(–1.591, –0.012)	0.542†	(–0.031, 1.115)

Having any health insurance	0.372	(–0.110, 0.854)	–0.232	(–0.698, 0.234)

Having a regular health care provider	0.010	(–0.394, 0.414)	0.163	(–0.196, 0.523)

Past medical history of cancer	0.594	(–0.528, 1.716)	0.066	(–0.412, 0.544)

Family history of cancer	0.189	(–0.213, 0.591)	0.073	(–0.220, 0.366)

Self-rated health status				

Excellent	Reference		Reference	

Very good	0.324	(–0.412, 1.059)	0.154	(–0.259, 0.567)

Good	0.006	(–0.738, 0.751)	0.436*	(0.005, 0.868)

Fair	0.124	(–0.726, 0.973)	0.223	(–0.279, 0.726)

Poor	0.675	(–0.402, 1.753)	–0.069	(–1.048, 0.909)

Instrumental variables				

Heard about test for lung cancer	0.697*	(0.174, 1.219)	-	

Heard about clinical trials	0.362†	(–0.015, 0.740)	-	

Constant	0.506	(–0.531, 1.542)	–1.005*	(–1.881, –0.129)

Correlation (*ρ*)			–0.689*	(–0.927, –0.060)

*Notes*: †p < 0.10, *p < 0.05, **p < 0.01. A set of dummy variables for survey regional division in the 2007 HINTS was included in the estimation but their coefficients are not presented here.

The correlation of the error terms (*ρ *= –0.689) was statistically significant (95% C.I.: –0.927, –0.060). Through statistical tests, the IVs used were found to be statistically significant determinants of HPV vaccine awareness but were not directly predictive of vaccine acceptability (results not shown).

## Discussion

This study found that HPV vaccine awareness had a significant positive impact on vaccine acceptability, a result not reported in previous studies which treated awareness as an exogenous variable in the equation for acceptability [21,26]. The deviation noted in this study suggests that disregarding the potential endogeneity of HPV vaccine awareness may possibly misinform policy makers in their efforts to promote HPV vaccine awareness. The statistically significant and negative correlation of the error terms (*ρ *= –0.689) indicates that unmeasured differences by HPV vaccine awareness (ε_2_) were correlated with unmeasured factors (ε_1_) that influenced HPV vaccine acceptability, rendering the estimation of coefficients in the probit model inconsistent. This negative correlation suggests that the unmeasured factors impact HPV vaccine awareness and acceptability in opposite directions. In other words, the marginal effectiveness of increasing awareness of the HPV vaccine on vaccine acceptability at the population level may be much greater than the observed difference between those who are aware of the HPV vaccine and those who are not.

Although caution must be exercised in interpreting the relationship between HPV vaccine awareness and the eventual initiation of the vaccination series in this study, awareness should still be recognized as an important starting point of a continuum that leads to adopting change. The four stages of HPV vaccine adoption described by Allen et al. (2009) include pre-contemplation, contemplation, preparation and action [32], fairly similar to the stages of behavior change constructs of the transtheoretical model earlier presented by Prochaska and colleagues (2008)[33]. Women in the pre-contemplation stage were generally unaware of the HPV vaccine; compared with women in the more advanced stages of change, women in pre-contemplation had the lowest median scores for knowledge of HPV, perceived severity of an HPV infection, and perceived benefits of the HPV vaccine - all of which are constructs of psychosocial models that predict vaccination behavior [25]. The results of this study complement the findings of Allen et al. (2009) [32] which suggested that raising HPV vaccine awareness can result in positive changes in attitudes towards vaccine, which can lead to vaccine uptake. The positive consequences of being aware urge policy makers to intensify efforts to raise HPV vaccine awareness at the population level, through the maximum involvement of relevant channels and influential motivators such as health care providers.

While low vaccination rates have been previously reported in women from low-income and minority groups who are most at risk of HPV infection and cervical cancer, this study found weak evidence of disparities in HPV vaccine acceptability across ethnicities and socioeconomic status. This suggests that there are presumably other factors that hinder women - who would be, in fact, receptive of the vaccine - from eventual vaccination. These factors present women from socioeconomically disadvantaged groups with a greater obstacle. Perceived barriers to HPV vaccination are essentially multidimensional [34] and may be further understood within the context of the health behavior change constructs. Moreover, barriers to an individual's intention to adapt a health behavior (e.g., lack of awareness and low acceptability) may be different from the barriers preventing a person from truly carrying out an intention to perform a particular behavior (e.g., vaccine cost and accessibility) [34]. Thus, aside from increasing emphasis on raising vaccine awareness, policy makers also need to be mindful of other barriers that impede vaccine uptake (i.e., high vaccine costs and limited access to health care facilities that administer HPV vaccinations), and to eliminate such barriers especially amongst the socioeconomically disadvantaged population so as to reduce socioeconomic disparities in HPV vaccination rates.

Several limitations should be acknowledged in this study. First, the HINTS 2007 did not offer any insights on how respondents' acceptability of the HPV vaccine may change in view of perceived barriers such as the affordability of the vaccine. Second, while this study quantified the potential effectiveness of improved HPV vaccine awareness on acceptability at the population level, the results of this study cannot directly provide individual-level evidence of the effect of HPV vaccine awareness on acceptability. Third, the key results were sensitive to the definition of HPV vaccine acceptability; when acceptability was defined as including "Not sure/It depends" in addition to "Yes", the main results were not statistically significant. Fourth, the relatively low response rate in the HINTS may not have captured a representative population. Finally, the HINTS 2007 was conducted almost two years after FDA's approval of Gardasil^®^, and levels of vaccine awareness and acceptability may have changed over the past few years.

## Conclusions

Despite these limitations, this study suggests that raising HPV vaccine awareness among those who are not aware of the HPV vaccine in the US - and therefore least likely to uptake HPV vaccination - could substantially increase HPV vaccine acceptability at the population level. This finding provides additional support to strengthening public health programs that increase awareness and policy efforts that address barriers to HPV vaccination.

## List of abbreviations

HPV: human papillomavirus; HINTS: Health Information National Trends Survey.

## Competing interests

No financial disclosures were reported by the authors of this paper. The authors declare that they have no competing interests.

## Authors' contributions

YKD conceived the study, analyzed the data, and drafted and completed the manuscript. In addition to conducting the literature review on HPV awareness and acceptability, KYW helped in data analysis and the drafting and completion of the manuscript. All authors read and approved the final manuscript.

## Pre-publication history

The pre-publication history for this paper can be accessed here:

http://www.biomedcentral.com/1471-2458/12/31/prepub
